# Evaluation of the Hepatoprotective Efficacy of Bee Pollen and Bee Pollen Ethanolic Extract–Loaded Solid Lipid Nanoparticles Against Lead Acetate–Induced Hepatotoxicity in Male Wistar Rats

**DOI:** 10.1155/bmri/3824359

**Published:** 2026-01-30

**Authors:** Khashayar Sanemar, Reza Mahjub, Fatemeh Nouri, Mojdeh Mohammadi

**Affiliations:** ^1^ Department of Pharmacology and Toxicology, School of Pharmacy, Hamadan University of Medical Sciences, Hamadan, Iran, umsha.ac.ir; ^2^ Department of Pharmaceutics, School of Medicine, Hamadan University of Medical Sciences, Hamadan, Iran, umsha.ac.ir; ^3^ Department of Pharmaceutical Biotechnology, School of Medicine, Hamadan University of Medical Sciences, Hamadan, Iran, umsha.ac.ir

**Keywords:** bee pollen, hepatoprotective, lead acetate, oxidative stress, solid lipid nanoparticles

## Abstract

**Introduction:**

Bee pollen, a natural product rich in polyphenols, exhibits remarkable antioxidant, anti‐inflammatory, and hepatoprotective properties.

**Aim:**

This study was aimed at evaluating the hepatoprotective effects of solid lipid nanoparticles (SLNs) loaded with bee pollen.

**Materials and Methods:**

SLNs were formulated and optimized by varying surfactant ratios and lipid contents at two different temperatures.

**Results and Discussion:**

The optimized bee pollen SLNs demonstrated a particle size of 118.6 nm, a PdI of 0.35, a zeta potential of −22.6 mV, and an entrapment efficiency of 92.7%. The in vitro release study showed minimal release during the initial 120 min, followed by a continuous increase up to 48 h, indicating a sustained and prolonged release profile. Both bee pollen and bee pollen ethanolic extract–loaded SLNs exhibited significant cytoprotective effects against lead‐induced cytotoxicity in HepG2 cells. In vivo studies revealed that treatment with bee pollen and especially bee pollen SLNs substantially ameliorated lead‐induced hepatic injury. Treatment notably reduced serum levels of aspartate aminotransferase, alanine aminotransferase, alkaline phosphatase, lactate dehydrogenase, malondialdehyde, and nitric oxide. Additionally, it enhanced the activity of glutathione peroxidase, catalase, superoxide dismutase, and total antioxidant capacity, as well as levels of total thiol, reduced glutathione, and liver tissue proteins. Histopathological analysis further confirmed that treatment, particularly with bee pollen SLNs, significantly improved lead‐induced hepatic structural damage.

**Conclusion:**

These findings confirm that bee pollen, and more prominently its SLN formulation, possesses strong hepatoprotective potential.

## 1. Introduction

Due to their long biological half‐lives and persistent detrimental effects on biological systems, exposure to toxic heavy metals has become a serious and critical global health concern [1–3]. Today, industrial waste, air pollutants, and contamination of soil, food, and water are recognized as the primary sources of lead (Pb) exposure [3–5]. Pb, as a toxic heavy metal, induces a wide range of behavioral, biochemical, and physiological adverse effects in humans. It is considered a potent systemic poison that causes oxidative damage in vital organs such as the brain, liver, heart, kidneys, and gastrointestinal tract (GIT), along with negative impacts on the immune, reproductive, and excretory systems, as well as erythrocytes [6–8]. The pathogenesis of Pb exposure is largely attributed to the induction of oxidative stress via the generation of various reactive oxygen species (ROS), including hydroxyl radicals (OH•), superoxide anions (O_2_•^−^), hydrogen peroxide (H_2_O_2_), and peroxyl radicals (ROO•). These reactive species disrupt the prooxidant/antioxidant balance, leading to significant downregulation of key antioxidant enzymes such as glutathione transferase (GST), superoxide dismutase (SOD), catalase (CAT), and glutathione peroxidase (GPx), ultimately resulting in severe damage to proteins and DNA structures [1, 4]. Clinical symptoms of Pb intoxication include weight loss, loss of appetite, anemia, kidney failure, and damage to the heart and liver.

Apitherapy, the use of bee products for therapeutic purposes, has been utilized successfully in traditional medicine since ancient times. In recent years, its diverse therapeutic benefits for the treatment of conditions such as burns, wounds, gastrointestinal disorders, ulcers, and various cancers have gained the attention of researchers [9]. Bee pollen, a key component of apitherapy, is rich in amino acids, proteins, hormones, enzymes, carbohydrates, minerals, lipids, vitamins, phenolic compounds, and antioxidants. Specifically, bee pollen consists of approximately 22.7% protein, 25.7% carbohydrates (including fructose and glucose), 5.1% lipids (including linoleic acid, *γ*‐linolenic acid, arachidic acid, phospholipids, phytosterols, and *β*‐sitosterol), and around 1.6% polyphenolic compounds (such as flavonoids, phenolic acids, anthocyanins, tannins, leukotrienes, and catechins) [10]. However, its clinical application is limited due to its high lipophilicity and correspondingly low oral bioavailability [11]. Therefore, developing an efficient drug delivery system to enhance the pharmacokinetic profile of bee pollen is of great importance. Among current delivery strategies, nanoparticle‐based systems are widely explored due to their effectiveness in improving oral bioavailability [12–14]. Recently, silver nanoparticles synthesized from bee pollen extract have demonstrated enhanced anticancer and antibacterial properties [15]. Additionally, chitosan‐based bee pollen formulations have been developed to improve its antibacterial and antioxidant activity [16].

Solid lipid nanoparticles (SLNs) are spherical particles typically less than 1000 nm in diameter, composed mainly of solid lipids, surfactants, solvents, and active pharmaceutical ingredients. Also known as lipospheres, SLNs can encapsulate both hydrophilic and lipophilic compounds and are regarded as promising nanocarriers for controlled drug delivery. Their notable features include biocompatibility, biodegradability, high stability, low toxicity, controlled release capabilities, protection of active compounds, and cost‐effective production. SLNs offer an effective platform for the delivery of poorly absorbed lipophilic compounds via the GIT.

The aim of this study was to prepare and characterize SLNs containing bee pollen extract and to evaluate their in vivo therapeutic efficacy in alleviating symptoms associated with Pb‐induced hepatitis. Additionally, the cytoprotective effects of the prepared nanoparticles were assessed in HepG2 cell lines.

## 2. Materials and Methods

### 2.1. Materials

Raw bee pollen granules were purchased from a local commercial beekeeper in the Alvand mountains (Hamadan Province, Iran). The pollen was stored in a dry, dark environment at 4°C until further use. Lead acetate (Pb(CH_3_COO)_2_), MTT (3‐[4,5‐dimethylthiazol‐2‐yl]‐2,5‐diphenyltetrazolium bromide), soy lecithin, Tween 80, glycerol monostearate (GMS), cellulose dialysis membrane (MWCO: 12,000 Da), quercetin, Folin–Ciocalteu reagent, ketamine, and xylazine were obtained from Sigma (St. Louis, MO, United States). Reagents including KH_2_PO_4_, NaOH, glacial acetic acid, Tris‐HCl, TCA, TBA, TPTZ, FeCl_3_·6H_2_O, FeSO_4_·7H_2_O, HCl, ethanol, DMSO, PBS, and AlCl_3_ were supplied by Merck‐Millipore (Darmstadt, Germany). DMEM, FBS, penicillin–streptomycin, and trypsin were provided by Gibco (United Kingdom). Ultrapure deionized water was prepared using a Milli‐Q system (Merck‐Millipore).

### 2.2. Extraction and Determination of Bee Pollen Flavonoids

Ethanolic extraction was carried out following a previously described protocol [12]. Briefly, bee pollen was ground using a mortar and pestle and macerated in 96% ethanol for 3 days at room temperature under magnetic stirring (Heidolph, Germany). The extract was filtered through Whatman paper and stored at 4°C.

Flavonoid content was measured colorimetrically using aluminum chloride and quercetin as standards [13]. A 2 mL aliquot of the extract was mixed with 1 mL of 5% (*w*/*v*) aluminum chloride in ethanol and incubated for 30 min. Absorbance was measured at 425 nm using a UV–Vis spectrophotometer (Specord 210 Plus, Analytik Jena, Germany). A calibration curve (*R* ^2^ = 0.9986) was plotted using quercetin (1–100 *μ*g/mL).

### 2.3. Preparation of the SLNs

SLNs were prepared using a modified solvent emulsification–evaporation method [14]. The organic phase consisted of GMS (50 mg) and soy lecithin, dissolved in 10 mL of ethanolic bee pollen extract, heated to 50°C, and sonicated. The aqueous phase contained Tween 80 in 20 mL of distilled water, preheated to 50°C. The organic phase was added dropwise to the aqueous phase under high‐speed homogenization (12,000 rpm, Heidolph, Germany) and stirred for 20 min. Ethanol was removed using a rotary evaporator at 30°C under vacuum, and the resulting suspension was cooled in an ice bath for 15 min. SLNs were collected by centrifugation (15,000 rpm, 30 min, 4°C). The supernatant was used for indirect calculation of entrapment efficiency (EE%) and LE%. Blank SLNs were prepared similarly using ethanol instead of bee pollen extract.

### 2.4. Determination of Physicochemical Properties of SLNs

SLNs were resuspended in distilled water. Size and polydispersity index (PdI) were measured via dynamic light scattering (DLS), and zeta potential was determined using laser Doppler anemometry (Nano ZS90, Malvern, United Kingdom), all at 25°C and in triplicate.

EE% and LE% were calculated based on the amount of unencapsulated flavonoids in the supernatant using the colorimetric method. Equations:

(1) EE%= Total Flavonoid content−free FlavonoidsTotal Flavonoid content∗100.

(2) LE%=Total Flavonoid content−free FlavonoidWeight of the nanoparticles∗100.

### 2.5. Freeze Drying Process of Nanoparticles

SLNs were resuspended in distilled water containing 2% (*w*/*v*) mannitol as a cryoprotectant, then lyophilized using a freeze dryer (Operon, South Korea). After freeze drying, the powder was rehydrated and reevaluated for size, PdI, and zeta potential.

### 2.6. Morphological Studies

SEM was used to assess nanoparticle morphology. Lyophilized SLNs were mounted on aluminum stubs, gold‐coated (24 mA, 120 s), and imaged using a scanning electron microscope (JEOL‐JSM‐6360, Japan) at 15 kV.

### 2.7. In Vitro Drug Release

Flavonoid release was assessed using a dialysis method [16]. Freeze‐dried SLNs (equivalent to 5 mg quercetin) were resuspended in simulated intestinal fluid (SIF, pH 6.8) and sealed in a dialysis bag (MWCO 12,000 Da), which was placed in 250 mL of SIF at 37°C and 150 rpm. At predetermined intervals, 2 mL samples were withdrawn and replaced with fresh medium. Flavonoid content was analyzed colorimetrically, and cumulative release was plotted over time.

### 2.8. Cell Culture

HepG2 cells were obtained from the Pasteur Institute of Iran and cultured in DMEM supplemented with 10% FBS, 100 U/mL penicillin, 30 *μ*g/mL streptomycin, and 20 *μ*g/mL gentamicin at 37°C and 5% CO_2_. Cells at 80% confluence (Passages 25–45) were used in experiments.

### 2.9. Cell Cytotoxicity Studies

MTT assay was used to assess cytotoxicity. Cells were seeded in 96‐well plates (1 × 10^4^ cells/well), incubated overnight, and treated with bee pollen extract, SLNs, or blank SLNs. After 24 h, 20 *μ*L of 5 mg/mL MTT was added, followed by 4 h incubation. The formazan crystals were dissolved in 180 *μ*L DMSO, and absorbance was read at 570nm.

### 2.10. Animal Experiment

The protocol was approved by the Ethics Committee of Hamadan University (IR.UMSHA.REC.1401.596). Male Wistar rats (180–220 g) were housed under standard conditions. Animals were divided into eight groups (*n* = 5):

Group I (negative control): oral olive oil + IP saline.

Group II (positive control): oral saline + IP Pb(CH_3_COO)_2_ (25 mg/kg/day, 7 days).

Groups III–V: oral bee pollen extract (200, 400, and 800 mg/kg) + IP Pb(CH_3_COO)_2_.

Groups VI–VIII: oral bee pollen SLNs (200, 400, and 800 mg/kg) + IP Pb(CH_3_COO)_2_.

On Day 15, rats were anesthetized (ketamine/xylazine) and sacrificed. Blood was collected, and liver tissues were harvested for biochemical and histological analysis.

#### 2.10.1. Determination of Serum Biochemical Parameters

ALT, AST, ALP, and LDH were quantified using commercial kits (Pars Azmoon Co., Iran).

#### 2.10.2. Measurement of Blood Lead Levels (BLLs)

Pb^2+^ concentration was measured using atomic absorption spectrometry (AAS, Hitachi Z‐5000, Japan).

#### 2.10.3. Determination of Liver Biochemical Parameters

Liver tissue (100 mg) was homogenized in PBS and centrifuged. Supernatants were assayed for SOD, CAT, GPx, glutathione (GSH), malondialdehyde (MDA), total thiol, and iNOS using ELISA kits (Navand Salamat Co., Iran).

#### 2.10.4. Determination of Liver Protein Levels

Bradford assay was used. Each well received 5 *μ*L of sample and 250 *μ*L of reagent, incubated for 15 min, and absorbance was read at 595 nm.

#### 2.10.5. Histopathological Analysis

Livers were fixed in 10% formalin, embedded in paraffin, sectioned (5 *μ*m), stained with H&E, and examined microscopically. Damage was scored (0–3) based on degeneration, vascular congestion, sinusoidal dilation, and hemorrhage.

### 2.11. Statistical Analysis

All data are presented as mean ± SD. Analyses were conducted using SPSS 19.0 (SPSS Inc., United States). Independent *t*‐tests were used for comparisons between two groups, and one‐way ANOVA followed by Tukey′s post hoc test was used for multiple group comparisons. A *p* value < 0.05 was considered statistically significant.

## 3. Results

### 3.1. Determination of Total Flavonoid Contents of Bee Pollen Extract

The total flavonoid content of the bee pollen extract was determined using the previously described colorimetric method. The total flavonoid content was approximately 12.4 ± 3.1 mg/g of bee pollen, based on quercetin equivalents.

### 3.2. Preparation and Characterization of SLNs

As described earlier, bee pollen–loaded SLNs were prepared via the emulsification and solvent evaporation method. The obtained nanoparticles were characterized for particle size, PdI, and zeta potential. The characterization results for different formulations are summarized in Tables 1 and 2.

**Table 1:** The size and PdI parameters at 50°C temperature.

**GMS/lecithin**	**Tween**	**Size (** **m** **e** **a** **n** ± **S** **D** **)**	**PdI (** **m** **e** **a** **n** ± **S** **D** **)**
0.25	1.0%	141.05 ± 94.64	0.63 ± 0.18
0.33	1.0%	100.4 ± 3.35	0.32 ± 0.01
0.5	1.0%	85.46 ± 8.55	0.51 ± 0.11
0.75	1.0%	64.86 ± 21.24	0.48 ± 0.07
1.0	1.0%	42.88 ± 1.28	0.46 ± 0.07
1.25	1.0%	62.95 ± 7.64	0.0
1.5	1.0%	89.17 ± 38.12	0.39 ± 0.06
1.75	1.0%	61.1 ± 16.2	0.41 ± 0.07
2.0	1.0%	149.4 ± 69.94	0.39 ± 0.09
3.0	1.0%	70.2 ± 13.1	0.31 ± 0.07
4.0	1.0%	107 ± 44.8	0.3 ± 0.1
1.0	0.5%	76 ± 0.5	0.5 ± 0.08
1.0	1.0%	63.2 ± 8.1	0.46 ± 0.005
1.0	1.5%	51.6 ± 4.7	0.49 ± 0.005
1.0	2.0%	21.95 ± 7.37	0.5 ± 0.12
1.0	2.5%	310.4 ± 25.74	0.24 ± 0.0
1.0	3.0%	243.55 ± 71.54	0.45 ± 0.17

**Table 2:** The size and PdI parameters at 70°C temperature.

**GMS/lecithin**	**Tween**	**Size (** **m** **e** **a** **n** ± **S** **D** **)**	**PdI (** **m** **e** **a** **n** ± **S** **D** **)**
0.25	1.0%	111.7 ± 26.1	0.54 ± 0.05
0.33	1.0%	77.15 ± 16.24	0.53 ± 0.12
0.5	1.0%	76.2 ± 8.7	0.39 ± 0.14
0.75	1.0%	75.4 ± 1.6	0.38 ± 0.11
1.0	1.0%	51.36 ± 6.52	0.37 ± 0.0
1.25	1.0%	71.15 ± 9.25	0.33 ± 0.05
1.5	1.0%	73.65 ± 9.44	0.32 ± 0.03
1.75	1.0%	76.05 ± 2.05	0.3 ± 0.16
2.0	1.0%	77.98 ± 11.09	0.31 ± 0.06
3.0	1.0%	83.41 ± 3.1	0.25 ± 0.01
4.0	1.0%	92.3 ± 0.7	0.37 ± 0.06
1.0	0.5%	232.4 ± 28.1	0.24 ± 0.005
1.0	1.0%	144.45 ± 52.05	0.43 ± 0.13
1.0	1.5%	115.1 ± 2.94	0.41 ± 0.01
1.0	2.0%	110.6 ± 11.4	0.4 ± 0.19
1.0	2.5%	118.6 ± 8.5	0.35 ± 0.1
1.0	3.0%	77.75 ± 64.34	0.44 ± 0.06

#### 3.2.1. Particle Size

As shown in Tables 1 and 2, the particle size of the SLNs ranged from 21.9 ± 7.37 nm to 310.4 ± 25.79 nm, confirming their nanometric scale. At 50°C, particle size decreased consistently with increasing lipid ratio (GMS/lecithin) from 0.25 to 1.25 and with an increase in Tween 80 concentration from 0.5% to 2.0%. At 70°C, particle size slightly decreased with increasing lipid ratio from 0.25 to 1.0, and a more notable decrease was observed with Tween 80 concentrations from 0.5% to 2.0%. The mean particle size of the optimized bee pollen SLNs before lyophilization was 118.6 ± 8.5 nm.

#### 3.2.2. PdI

According to Tables 1 and 2, the PdI values ranged from 0.24 ± 0.0 to 0.63 ± 0.18. At 50°C, PdI decreased gradually with an increasing lipid ratio (GMS/lecithin) from 1.75 to 4.0, while no clear trend was observed for Tween 80 concentration. At 70°C, PdI significantly decreased with increasing lipid ratio from 0.25 to 1.75 and also with Tween 80 concentrations from 1.5% to 2.5%. The PdI of the optimized formulation prior to lyophilization was 0.35 ± 0.1.

### 3.3. Entrapment Efficacy Studies

EE% values for the bee pollen SLNs, prepared with different lipid and surfactant ratios at 50°C and 70°C, are shown in Tables 3 and 4. The EE% ranged from 56.7*%* ± 9.5*%* to 92.7*%* ± 2.9*%* across different formulations. A significant increase in EE% was observed with increasing preparation temperature.

**Table 3:** Entrapment efficacy of different optimized bee pollen ethanolic extract–loaded solid lipid nanoparticles at a temperature of 50°C.

**Number**	**GMS/lecithin**	**Tween**	**% entrapment (** **m** **e** **a** **n** ± **S** **D** **)**
1	0.33	1.0%	63.1*%* ± 2.2%
2	1.5	1.0%	64.8*%* ± 6.3%
3	3.0	1.0%	68.2*%* ± 1.7%
4	4.0	1.0%	56.7*%* ± 9.5%

**Table 4:** Entrapment efficacy of different optimized bee pollen ethanolic extract–loaded solid lipid nanoparticles at a temperature of 70°C.

**Number**	**GMS/lecithin**	**Tween**	**% entrapment (** **m** **e** **a** **n** ± **S** **D** **)**
1	0.25	1.0%	75.4*%* ± 2.6%
2	1.0	1.0%	71.5*%* ± 0.8%
3	1.75	1.0%	79.6*%* ± 1.4%
4	3.0	1.0%	75.4*%* ± 8.1%
5	1.0	2.0%	91.9*%* ± 3.1%
6	1.0	2.5%	92.7*%* ± 2.9%

### 3.4. Freeze Drying Process

The particle size, PdI, and zeta potential of the optimized SLNs were analyzed before and after lyophilization (*n* = 3). As shown in Table 5, lyophilization did not cause any significant change in the physicochemical characteristics of the optimized SLNs.

**Table 5:** The physicochemical parameters before and after the lyophilization process.

**Physicochemical properties**	**Before lyophilization**	**After lyophilization**
Size (nm) (mean ± SD)	118.6 ± 8.5	150.3 ± 3.2
Polydispersity index (PdI) (mean ± SD)	0.35 ± 0.1	0.39 ± 0.4
Zeta potential (mV) (mean ± SD)	−22.6 ± 3.3	−30.5 ± 4.8

### 3.5. In Vitro Drug Release Studies

**Figure 1:**
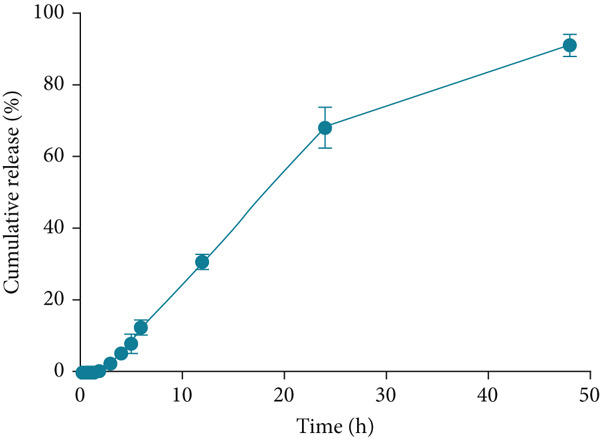
The percent of cumulative release of bee pollen ethanolic extract–loaded solid lipid nanoparticles (SLNs) during 48 h of experiment. Values are represented as mean ± SEM (*n* = 3).

The in vitro release profile of bee pollen flavonoids from SLNs was evaluated in SIF and is illustrated in Figure 1. Minimal release was observed during the first 2 h. However, a sustained release pattern was recorded afterward, with a cumulative release of 89.2*%* ± 1.26*%* at the 48‐h time point.

### 3.6. Morphological Studies (SEM)

**Figure 2:**
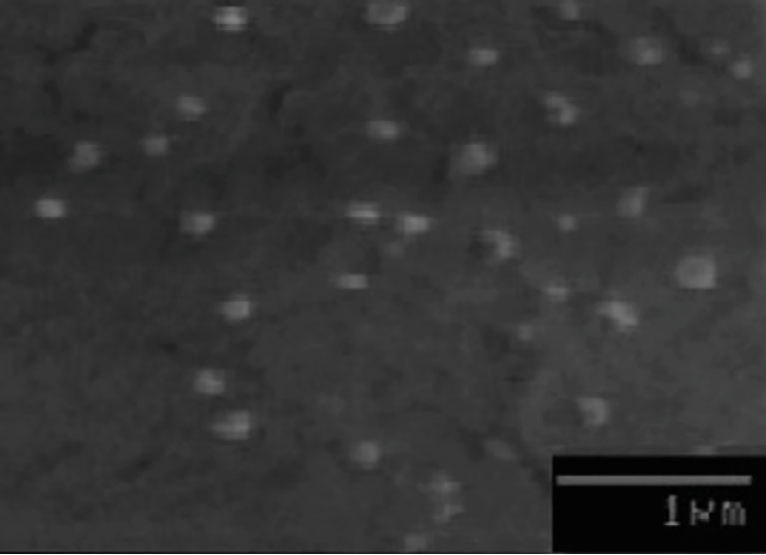
SEM images of the optimized bee pollen ethanolic extract–loaded solid lipid nanoparticles (20,000x).

SEM analysis was used to assess the morphology of lyophilized nanoparticles, as presented in Figure 2. The nanoparticles exhibited spherical to subspherical morphology without signs of aggregation. The particle sizes observed under SEM were in good agreement with the DLS results.

### 3.7. Cell Cytotoxicity Studies

Cytotoxic effects of bee pollen extract and bee pollen SLNs on HepG2 cells were examined using the MTT assay. Cells were treated with different concentrations (6.25–1600 *μ*g/mL) for 24 and 48 h. As illustrated in Figure 3a,b, no significant cytotoxicity was observed for either treatment up to 400 *μ*g/mL compared to the control. Figure 3c,d shows that Pb(CH_3_COO)_2_ exposure significantly reduced HepG2 cell viability in a dose‐dependent manner, with IC_50_ values of 20.44 and 10.41 *μ*g/mL after 24 and 48 h, respectively. Treatment with bee pollen extract or SLNs significantly restored cell viability in Pb‐exposed cells (Figure 3e,f), with the SLNs showing superior cytoprotective effects in a dose‐ and time‐dependent manner.

Figure 3The effects of lead acetate, bee pollen, and bee pollen SLNs on the cell viability of HepG2 cells during 24 and 48 h treatment. Data are expressed as the mean ± SD. (a, b) Viability of cells exposed to different doses of bee pollen and bee pollen SLNs during 24 and 48 h, respectively. (c, d) Viability of cells exposed to different concentrations of lead acetate during 24 and 48 h, respectively. (e, f) Cytoprotective effects of bee pollen and bee pollen SLNs on HepG2 cells exposed to lead acetate during 24 and 48 h, respectively. Significant differences are expressed as ^∗∗∗^ *p* < 0.001, ^∗∗^ *p* < 0.01, and ^∗^ *p* < 0.05 between lead acetate and different treatment groups and ^@@@^ *p* < 0.001 between bee pollen SLNs and bee pollen groups.

**Figure:**
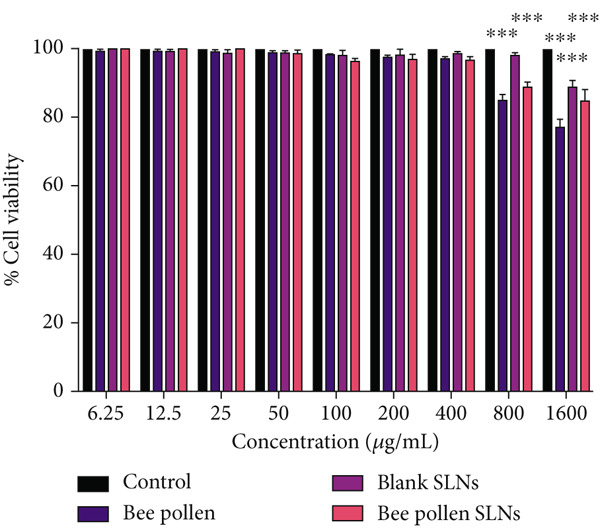
(a)

**Figure:**
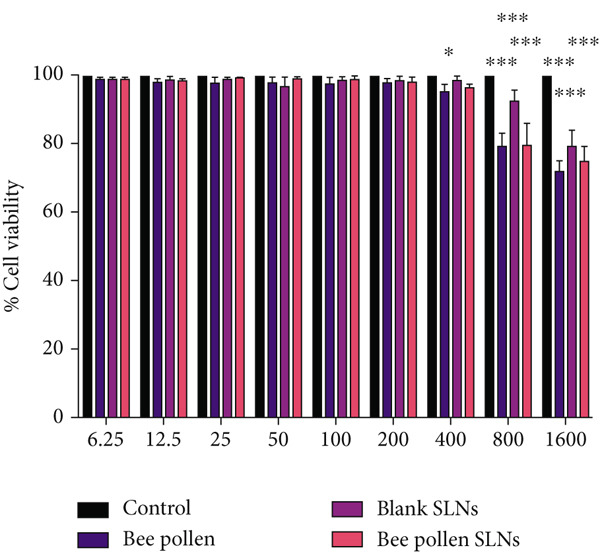
(b)

**Figure:**
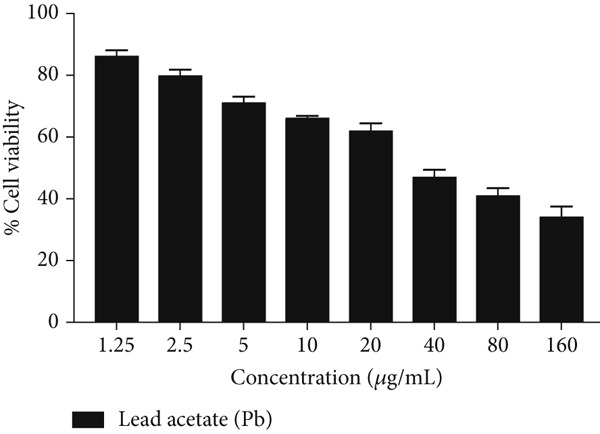
(c)

**Figure:**
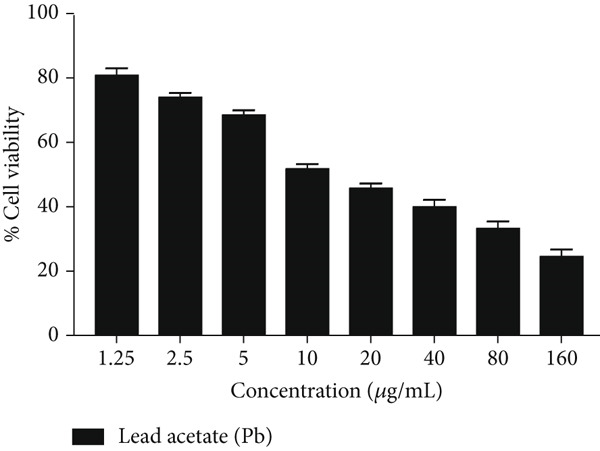
(d)

**Figure:**
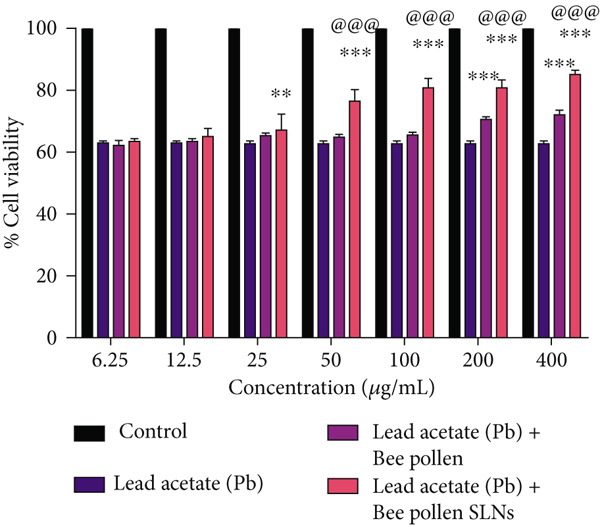
(e)

**Figure:**
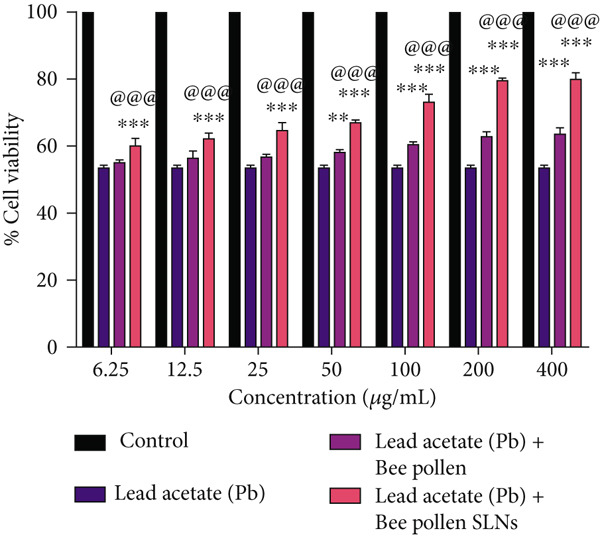
(f)

### 3.8. Effects of Bee Pollen and Bee Pollen SLNs on Serum Biochemical Parameters

Serum liver enzymes (AST, ALT, ALP, and LDH) were measured and are shown in Figure 4. Pb(CH_3_COO)_2_ administration significantly elevated all four enzymes compared to the control group (*p* < 0.001). Treatment with bee pollen or bee pollen SLNs significantly decreased these levels in a dose‐dependent manner (*p* < 0.01 or *p* < 0.001). Bee pollen SLNs were significantly more effective than bee pollen alone in reducing serum AST, ALT, ALP, and LDH activities at corresponding doses (*p* < 0.001).

Figure 4(a–d) The effects of lead acetate intoxication on liver function parameters (AST, ALT, ALP, and LDH) in different treatment groups. Each value represented as mean ± SEM (*n* = 5). Significant differences are expressed as ^###^ *p* < 0.001 between control and lead acetate group; ^∗∗∗^ *p* < 0.001, ^∗∗^ *p* < 0.01, and ^∗^ *p* < 0.05 between lead acetate and different treatment groups; and ^@@@^ *p* < 0.001 and ^@^ *p* < 0.05 between bee pollen SLNs and bee pollen groups.

**Figure:**
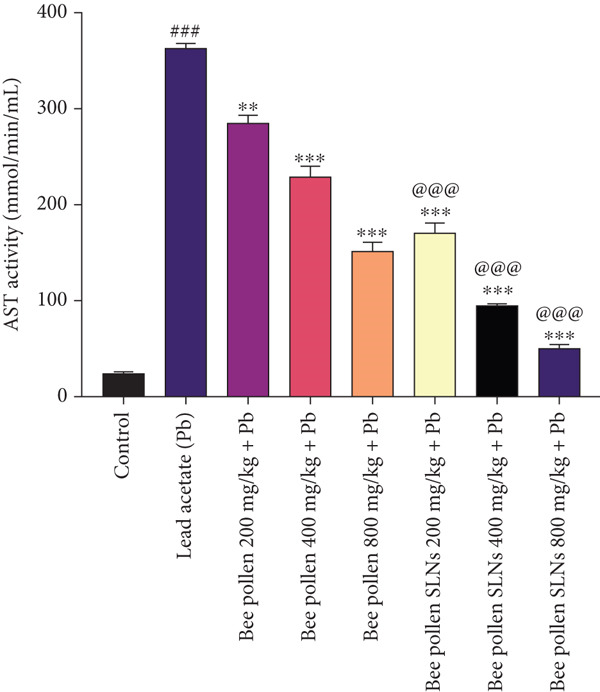
(a)

**Figure:**
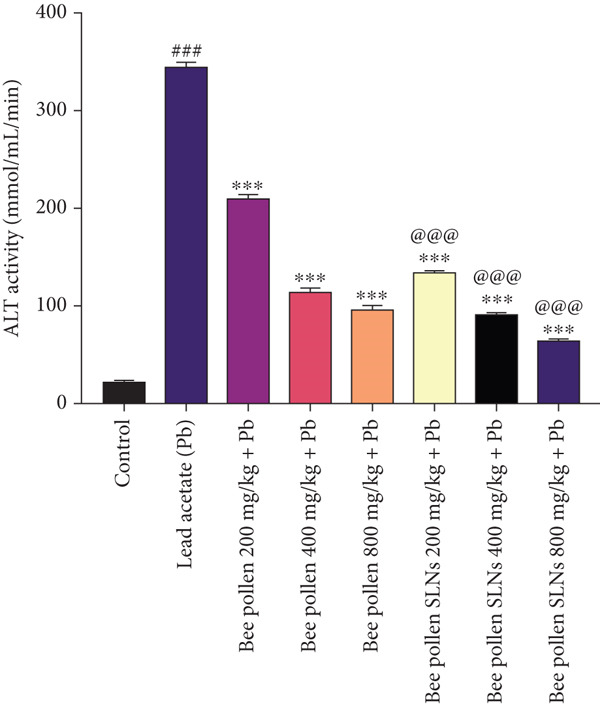
(b)

**Figure:**
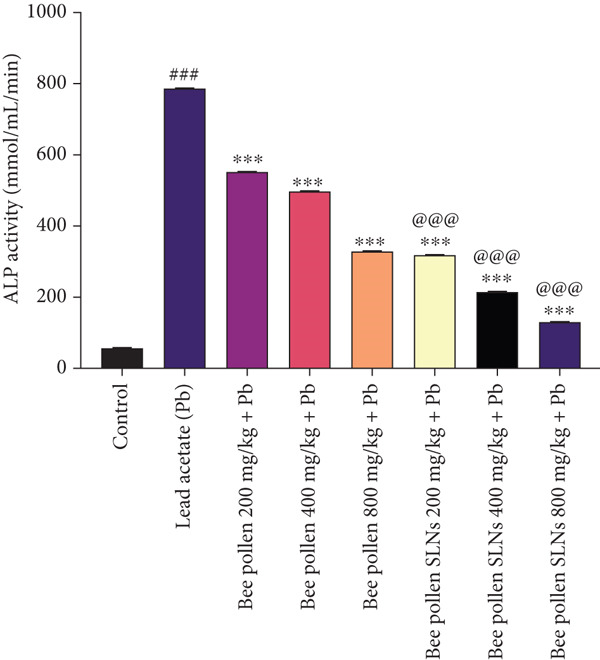
(c)

**Figure:**
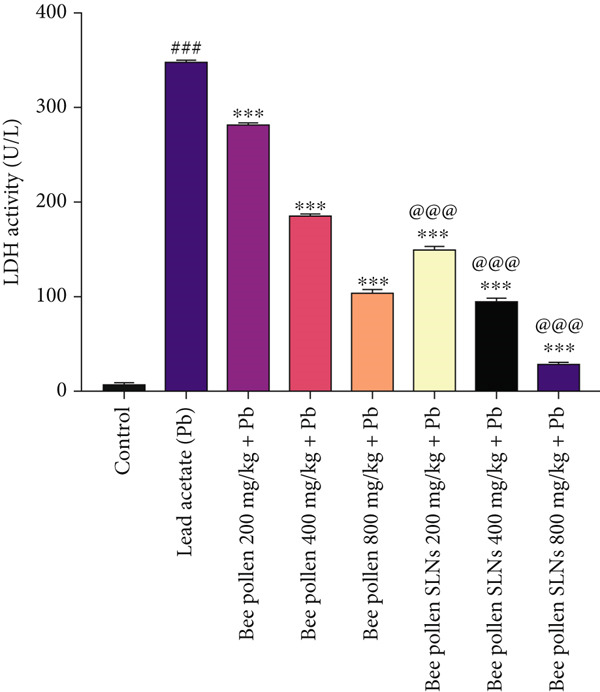
(d)

### 3.9. Effects of Bee Pollen and Bee Pollen SLNs on BLLs

**Figure 5:**
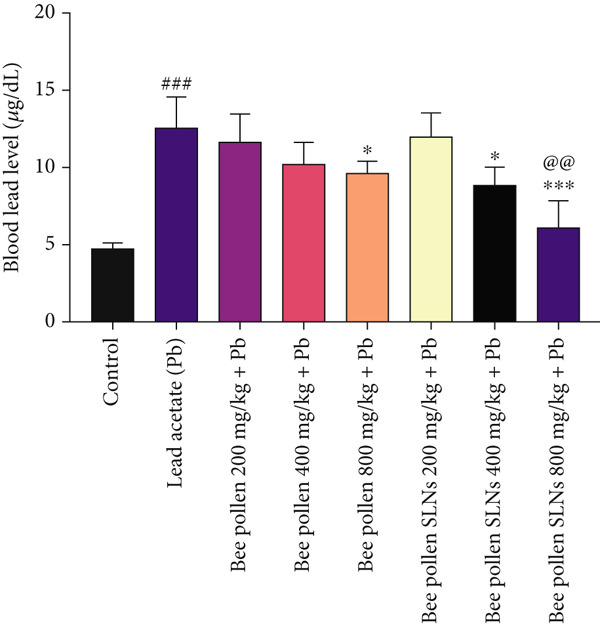
The effects of lead acetate intoxication on blood lead levels (BLL) in different treatment groups. Each value represented as mean ± SEM (*n* = 5). Significant differences are expressed as ^###^ *p* < 0.001 between control and lead acetate group, ^∗∗^ *p* < 0.001 and ^∗^ *p* < 0.05 between lead acetate and different treatment groups, and ^@@@^ *p* < 0.001 and ^@^ *p* < 0.05 between bee pollen SLNs and bee pollen groups.

The effect of treatments on BLLs is illustrated in Figure 5. Pb(CH_3_COO)_2_ significantly increased BLL compared to the control group (*p* < 0.001). Bee pollen at 800 mg/kg significantly reduced BLL (*p* < 0.05), while lower doses had no significant effect. Bee pollen SLNs at 400 and 800 mg/kg significantly decreased BLL compared to the Pb group (*p* < 0.05 and *p* < 0.001, respectively). No significant difference was observed between bee pollen and SLNs at 200 and 400 mg/kg, but SLNs at 800 mg/kg were significantly more effective than the equivalent dose of bee pollen extract (*p* < 0.01).

### 3.10. Effects of Bee Pollen and Bee Pollen SLNs on Liver Biochemical Parameters

As shown in Figure 6a, Pb(CH_3_COO)_2_ administration significantly increased MDA levels in liver tissue homogenates compared to the control group (*p* < 0.001). Treatment with bee pollen at various doses significantly reduced liver MDA levels compared to the Pb group (*p* < 0.05, *p* < 0.001, and *p* < 0.001, respectively). Similarly, bee pollen SLNs significantly decreased liver MDA levels at all tested doses (*p* < 0.001). Moreover, MDA levels in bee pollen SLNs groups were significantly lower than those in corresponding bee pollen groups at all doses (*p* < 0.001).

Figure 6(a–h) The effect of lead acetate intoxication on the liver oxidative stress biomarkers (MDA, NO, CAT, GPx, SOD, total thiol, GSH, and TAC) in different treatment groups. Each value represented as mean ± SEM (*n* = 5). Significant differences are expressed as ^###^ *p* < 0.001 between control and lead acetate group and ^@@@^ *p* < 0.001 and ^@@^ *p* < 0.01 between bee pollen SLNs and bee pollen groups.

**Figure:**
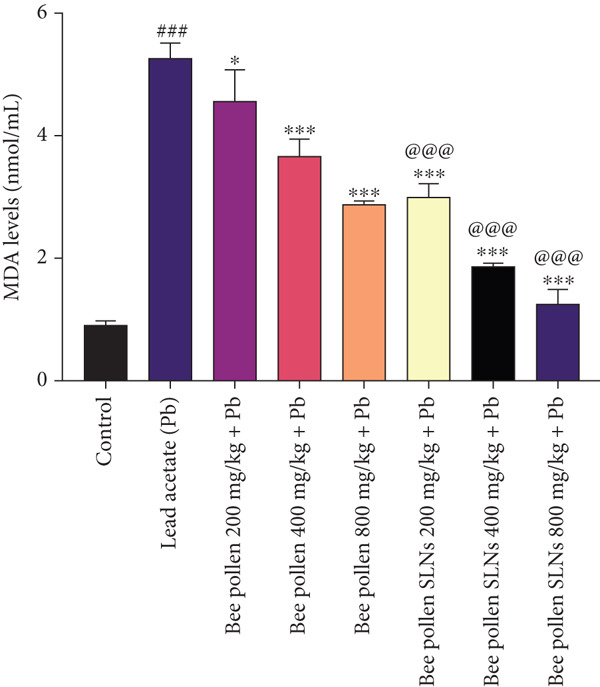
(a)

**Figure:**
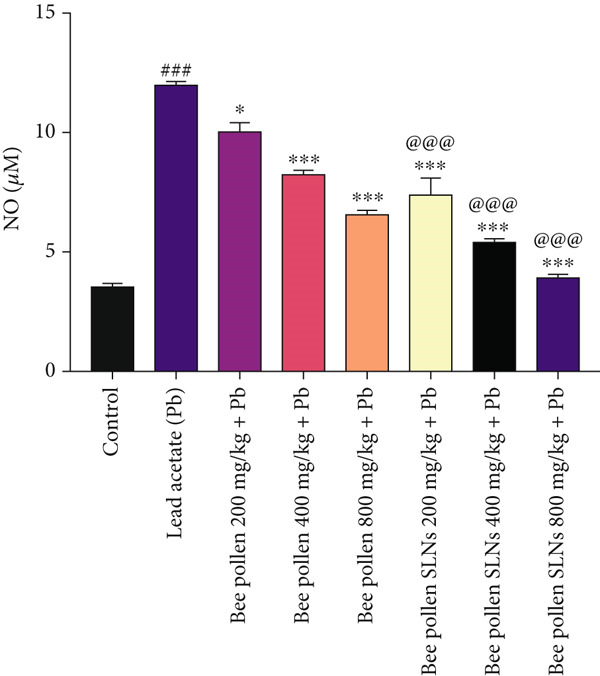
(b)

**Figure:**
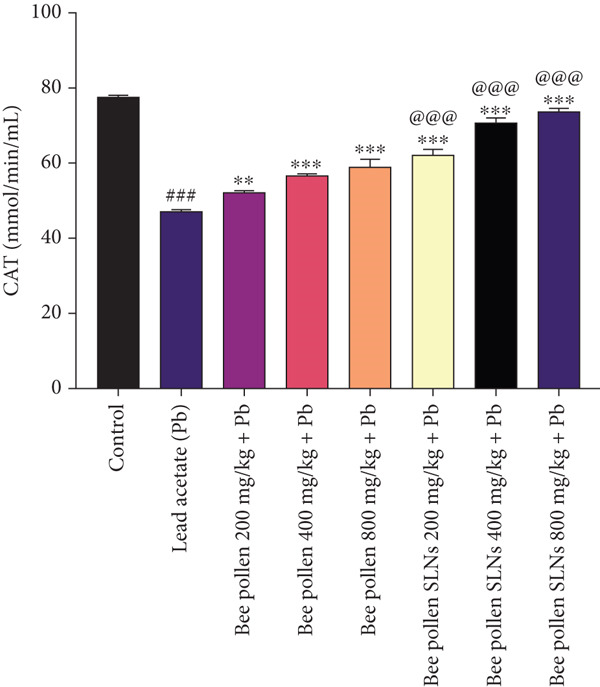
(c)

**Figure:**
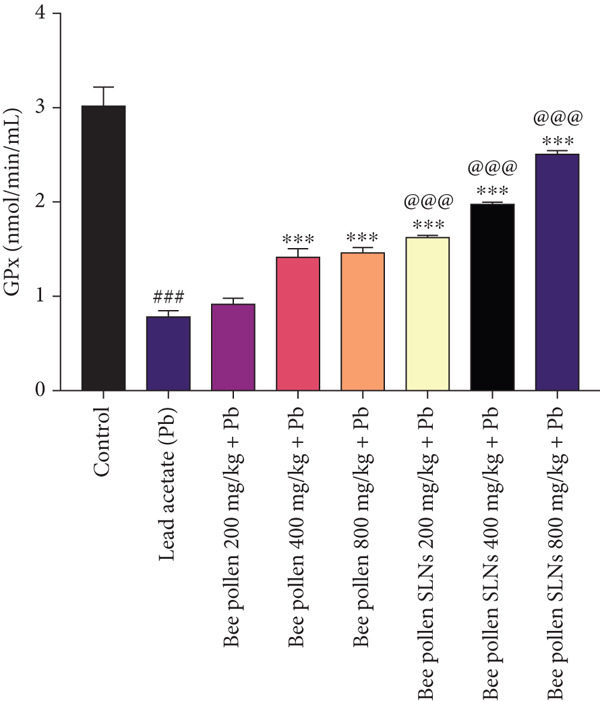
(d)

**Figure:**
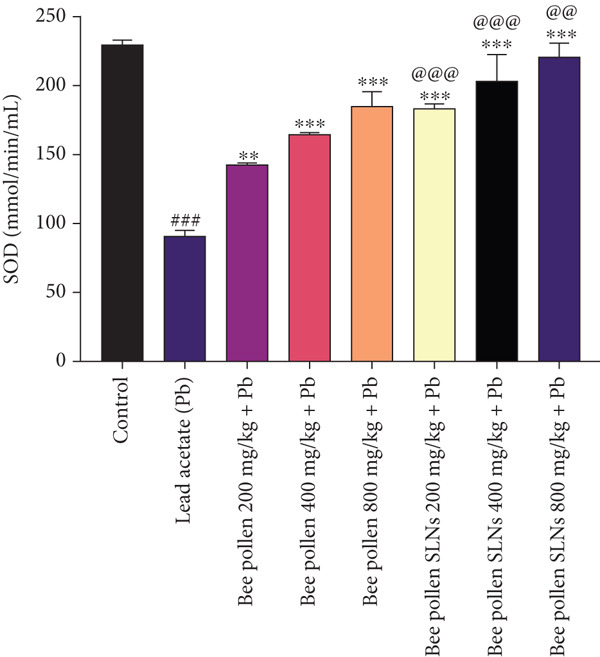
(e)

**Figure:**
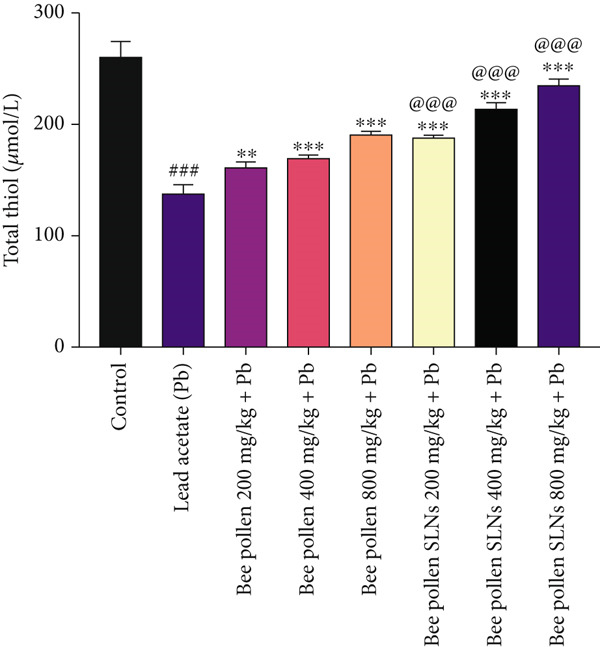
(f)

**Figure:**
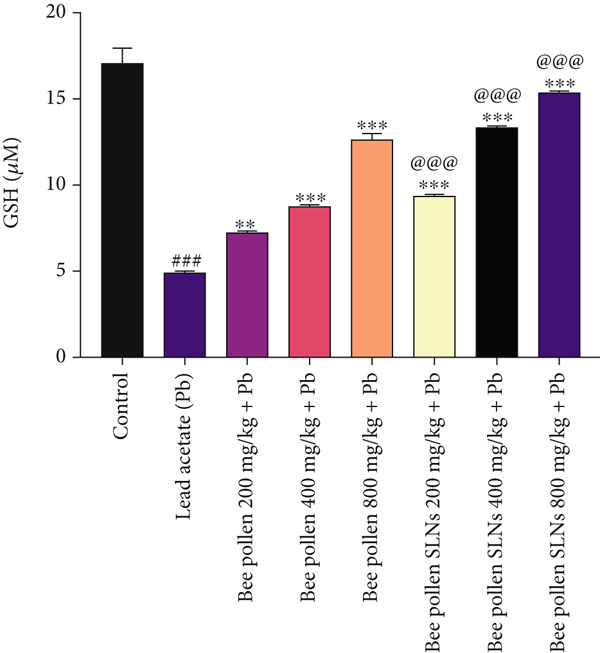
(g)

**Figure:**
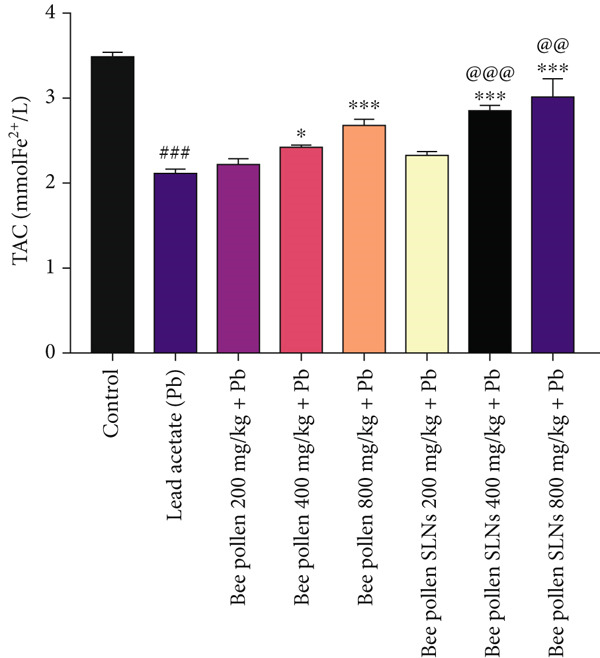
(h)

Figure 6b shows that Pb intoxication caused a significant elevation in liver nitric oxide (NO) levels compared to control (*p* < 0.001). Bee pollen treatment significantly reduced liver NO levels at all doses compared to Pb‐exposed rats (*p* < 0.05, *p* < 0.001, and *p* < 0.001, respectively). Bee pollen SLNs also significantly decreased NO levels (*p* < 0.001 across doses), with significantly greater reductions than bee pollen alone (*p* < 0.001).

As presented in Figure 6c, Pb exposure caused a significant decrease in total thiol content in liver homogenates versus control (*p* < 0.001). Treatment with bee pollen significantly restored total thiol levels (*p* < 0.01, *p* < 0.001, and *p* < 0.001, respectively), and bee pollen SLNs showed a more pronounced improvement (*p* < 0.001 for all doses). Total thiol content was significantly higher in bee pollen SLNs groups compared to bee pollen at equivalent doses (*p* < 0.001).

Figure 6d illustrates that GSH levels were significantly reduced by Pb intoxication compared to control (*p* < 0.001). Bee pollen treatment significantly increased liver GSH content (*p* < 0.01, *p* < 0.001, and *p* < 0.001, respectively), and bee pollen SLNs further enhanced GSH levels (*p* < 0.001 for all doses), with significantly higher restoration compared to bee pollen alone (*p* < 0.001).

Figure 6e shows a significant reduction in SOD activity after Pb exposure versus control (*p* < 0.001). Both bee pollen and bee pollen SLNs treatments significantly elevated SOD activity (bee pollen: *p* < 0.01, *p* < 0.001, and *p* < 0.001; SLNs: *p* < 0.001 across all doses). SLNs induced a significantly greater increase than bee pollen at all doses (*p* < 0.001, *p* < 0.001, and *p* < 0.01).

As depicted in Figure 6f, CAT activity was markedly decreased by Pb compared to control (*p* < 0.001). Bee pollen supplementation significantly improved CAT activity (*p* < 0.01, *p* < 0.001, and *p* < 0.001), and bee pollen SLNs further enhanced CAT activity (*p* < 0.001 for all doses), with SLNs showing significantly higher activity than bee pollen (*p* < 0.001).

Figure 6g demonstrates that Pb intoxication significantly decreased GPx activity (*p* < 0.001). Bee pollen at 200 mg/kg did not significantly alter GPx levels; however, doses of 400 and 800 mg/kg significantly increased GPx activity compared to Pb alone (*p* < 0.001). Bee pollen SLNs significantly enhanced GPx activity at all doses (*p* < 0.001), surpassing the effects of bee pollen (*p* < 0.001).

Figure 6h shows a significant decrease in total antioxidant capacity (TAC) in Pb‐exposed rats versus control (*p* < 0.001). Bee pollen at 200 mg/kg did not significantly alter TAC, while 400 and 800 mg/kg doses significantly improved TAC (*p* < 0.05 and *p* < 0.001). Similarly, bee pollen SLNs at 200 mg/kg had no significant effect, but doses of 400 and 800 mg/kg significantly increased TAC (*p* < 0.001). TAC was significantly higher in SLNs groups compared to bee pollen at these doses (*p* < 0.001 and *p* < 0.01).

### 3.11. Effects of Bee Pollen and Bee Pollen SLNs on Liver Protein Levels

**Figure 7:**
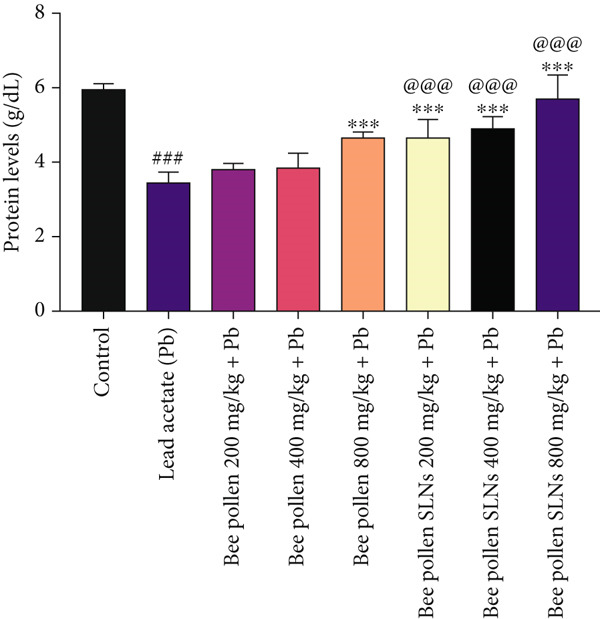
The effects of lead acetate intoxication on liver protein levels in different treatment groups. Each value represented as mean ± SEM (*n* = 5). Significant differences are expressed as ^###^ *p* < 0.001 between control and lead acetate group, ^∗∗∗^ *p* < 0.001 between lead acetate and different treatment groups, and ^@@@^ *p* < 0.001 between bee pollen SLNs and bee pollen groups.

As shown in Figure 7, Pb(CH_3_COO)_2_ significantly reduced liver protein levels compared to the control group (*p* < 0.001). Bee pollen treatment at 200 and 400 mg/kg did not significantly affect protein levels, whereas 800 mg/kg significantly increased liver protein compared to Pb alone (*p* < 0.001). All doses of bee pollen SLNs significantly elevated liver protein levels versus Pb‐exposed rats (*p* < 0.001). Moreover, protein levels in SLNs groups were significantly higher than those in corresponding bee pollen groups at all doses (*p* < 0.001).

### 3.12. Effects of Bee Pollen and Bee Pollen SLNs on Liver Histopathology

Histopathological changes following Pb intoxication and treatments are presented in Figure 8. Liver sections from the control group showed normal architecture with hepatocytes arranged in cords radiating from the central vein (CV) (Figure 8a). In contrast, the Pb‐intoxicated group displayed marked pathological changes including portal vein occlusion, hemorrhage, inflammation, congestion, hepatocyte necrosis, pyknotic nuclei, and fibrosis characterized by fibroblast accumulation (Figure 8b).

Figure 8The histopathological alterations that occurred in liver tissue in different treated groups. (a) Control group (olive oil), (b) lead acetate group (25 mg/kg), (c) bee pollen (200 mg/kg) + lead acetate, (d) bee pollen (400 mg/kg) + lead acetate, (e) bee pollen (800 mg/kg) + lead acetate, (f) bee pollen SLN (200 mg/kg) + lead acetate, (g) bee pollen SLN (400 mg/kg) + lead acetate, and (h) bee pollen SLN (800 mg/kg) + lead acetate.

**Figure:**
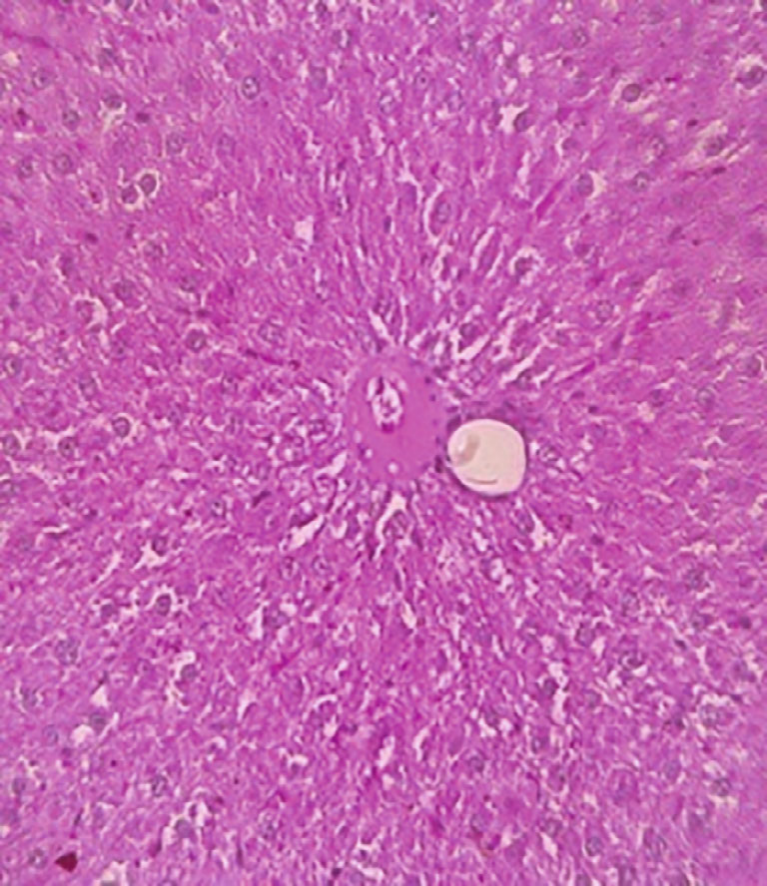
(a)

**Figure:**
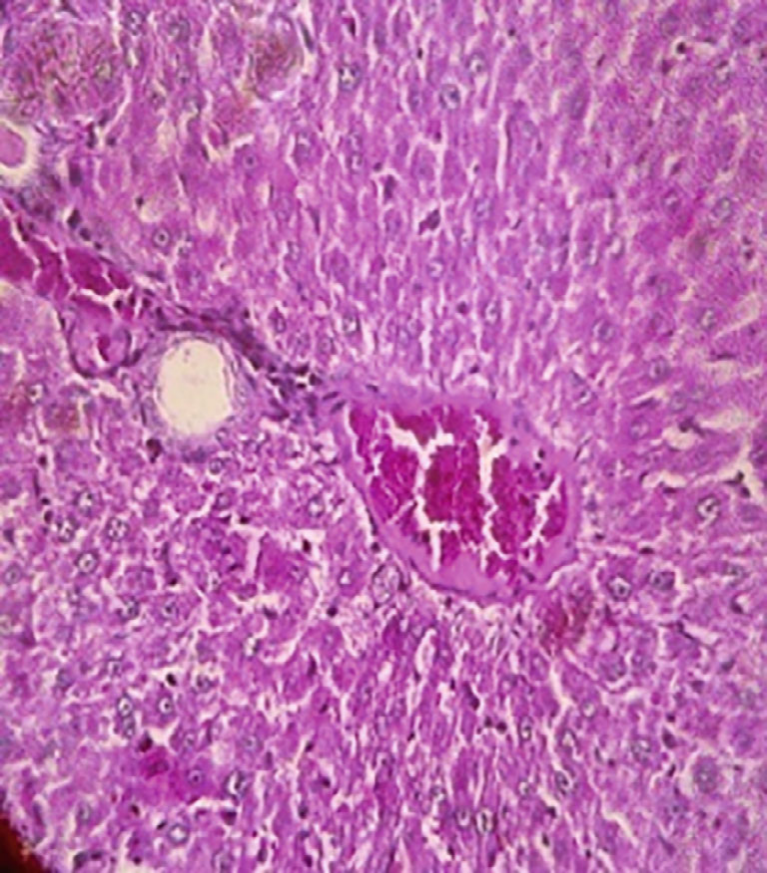
(b)

**Figure:**
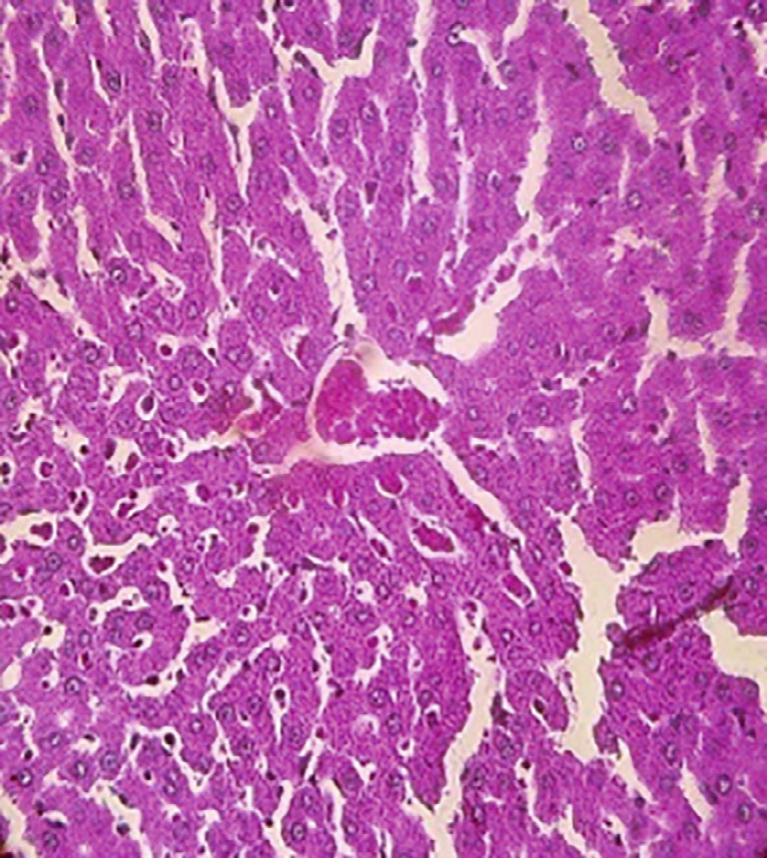
(c)

**Figure:**
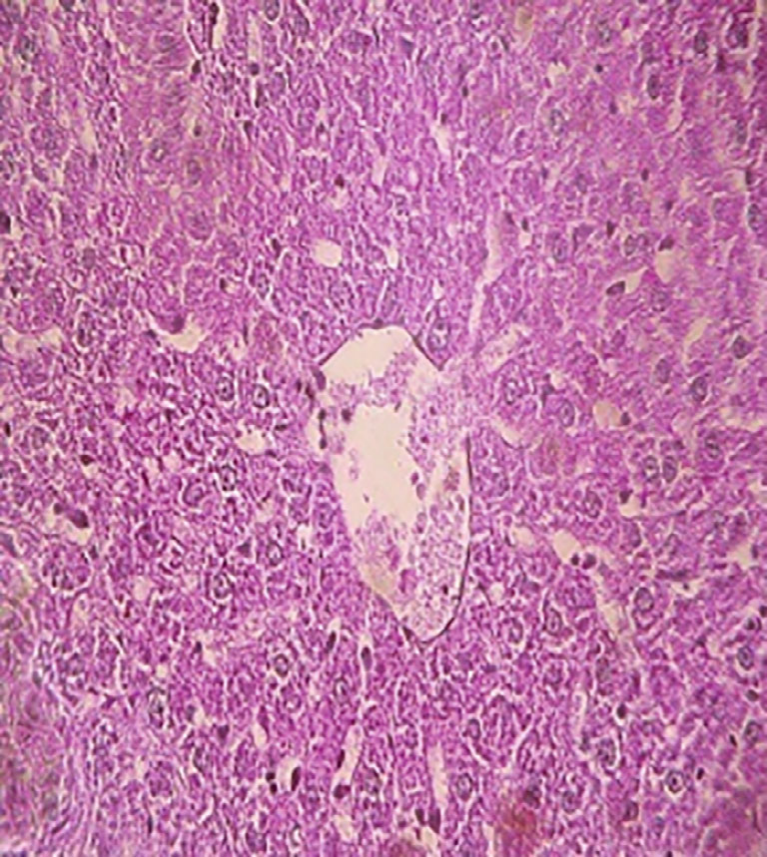
(d)

**Figure:**
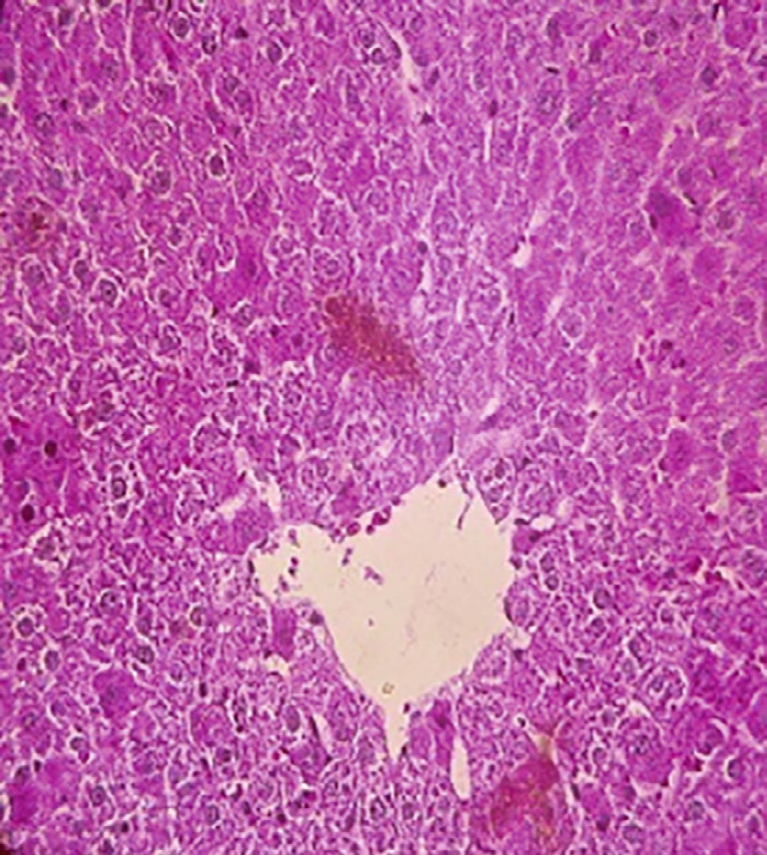
(e)

**Figure:**
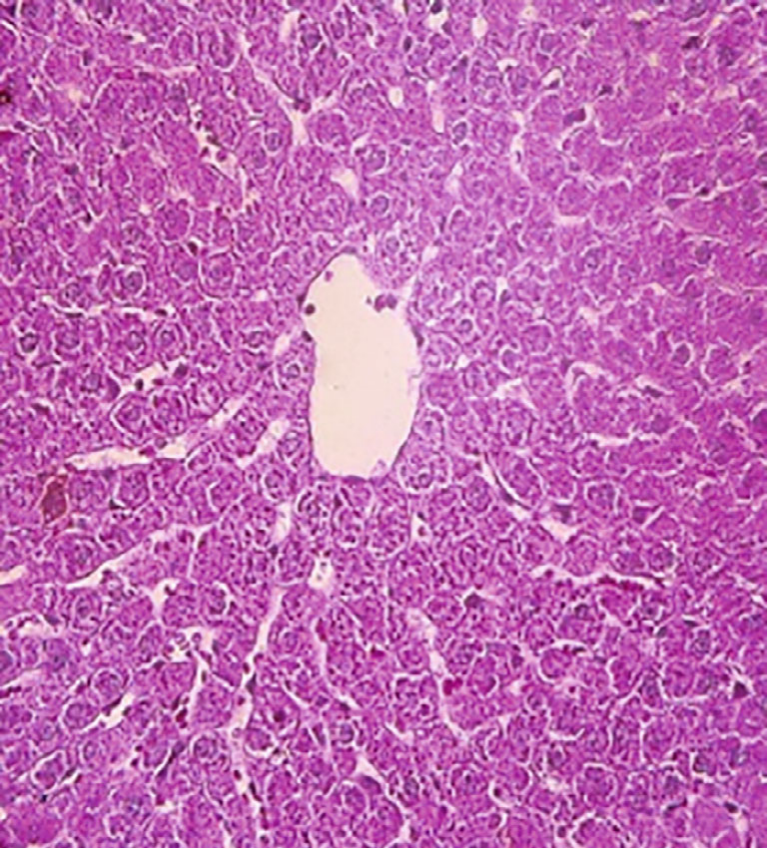
(f)

**Figure:**
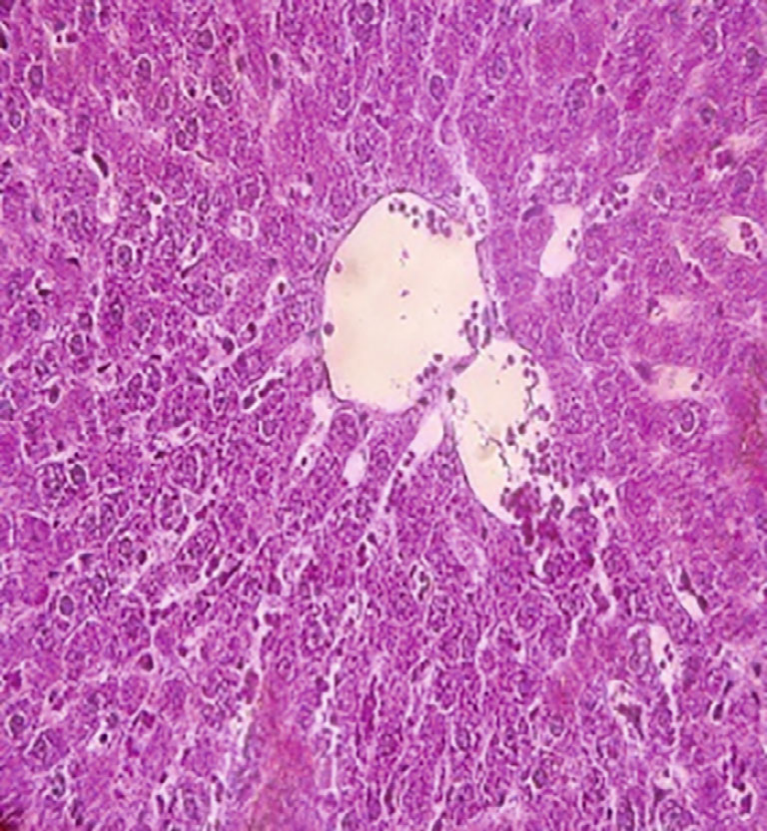
(g)

**Figure:**
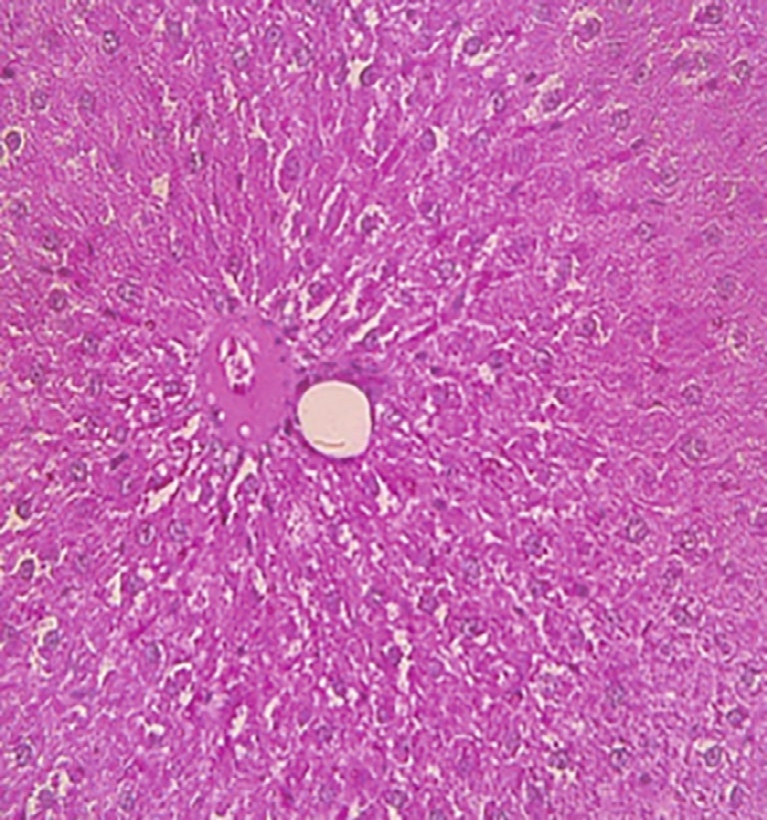
(h)

Cotreatment with bee pollen (200 mg/kg) partially ameliorated inflammation, occlusion, and congestion, but hemorrhage, necrosis, pyknotic nuclei, and fibrosis remained evident (Figure 8c). At 400 mg/kg, bee pollen reduced inflammation, hemorrhage, congestion, and portal vein occlusion; however, significant necrosis, fibrosis, and pyknotic nuclei persisted (Figure 8d). Treatment with 800 mg/kg bee pollen resulted in considerable improvement in inflammation, hemorrhage, congestion, and portal vein occlusion, although necrosis and fibrosis were still present (Figure 8e).

Groups receiving bee pollen SLNs (200 mg/kg) showed moderate tissue inflammation, hemorrhage, congestion, portal vein occlusion, necrosis, pyknotic nuclei, and fibrosis (Figure 8f). At 400 mg/kg, bee pollen SLNs significantly reduced inflammation, hemorrhage, congestion, and portal vein occlusion, with marked decreases in necrosis, pyknotic nuclei, and fibrosis (Figure 8g). Notably, the 800 mg/kg SLNs group exhibited near‐normal histology, with the disappearance of inflammation, hemorrhage, congestion, and portal vein occlusion. Fibrosis, necrosis, and nuclear pyknosis were markedly reduced, resembling control liver morphology (Figure 8h).

## 4. Discussion

With the advancement of science and increasing industrialization, human exposure to hazardous environmental substances such as heavy metals has significantly risen. Consequently, there is a growing need for preventive and therapeutic agents to mitigate the harmful effects of these pollutants [17]. Numerous studies have highlighted the therapeutic and ameliorative potential of natural antioxidants against toxic heavy metals, offering promising and valuable outcomes [3].

Bee pollen is a nutrient‐rich natural product containing high levels of amino acids, proteins, hormones, enzymes, carbohydrates, minerals, fats, vitamins, and phenolic compounds. It exhibits a broad spectrum of biological activities including antioxidant, anti‐inflammatory, anticancer, immunomodulatory, antibacterial, antifungal, hepatoprotective, and antiatherosclerotic effects [18]. Its antioxidant activity is primarily attributed to bioactive constituents such as flavonoids, phenolic acids, carotenoids, GSH, vitamin C, and vitamin E [12].

In recent years, nanotechnology applications in biological systems have attracted considerable attention. Efforts have been made to develop nanoparticles with high efficacy and minimal toxicity for drug and bioactive ingredient delivery [13]. Lipid‐based nanoparticles are particularly advantageous due to their lower toxicity, higher EE%, biocompatibility, and biodegradability compared to inorganic or polymeric nanoparticles. Among them, SLNs have emerged as promising nanocarriers, offering versatile administration routes such as oral, nasal, parenteral, rectal, and ocular delivery [14, 19, 20].

Considering these advantages and the oral bioavailability potential of SLNs, this study was aimed at designing, fabricating, and characterizing bee pollen ethanolic extract–loaded SLNs to enhance bioavailability and improve the hepatoprotective effects of bee pollen against Pb(CH_3_COO)_2_‐induced liver toxicity. Bee pollen, exhibiting low hydrophilicity, is a suitable candidate for encapsulation within lipid‐based nanosystems. Thus, SLNs were formulated with optimized parameters—minimal particle size, low PdI, and maximal EE%—to deliver bee pollen flavonoids effectively.

Lipid and surfactant composition play pivotal roles in determining physicochemical characteristics of SLNs, including particle size, PdI, zeta potential, EE%, and drug release profile [21]. Particle size is a critical factor in optimizing SLNs [22]. Our study demonstrated that increasing homogenization temperature led to decreased particle size, consistent with findings by Jenning and Gohla [23]. Similarly, Nasiri et al. reported a reduction in particle size with elevated aqueous phase temperature due to decreased suspension viscosity and increased kinetic energy, enhancing lipid droplet dispersion [24]. Surfactant concentration also significantly influences particle size distribution [25]. In this study, increasing Tween 80 concentration from 0.5% to 3.0% markedly reduced SLN size, corroborating Obinu et al.′s report of size reduction with higher surfactant levels [26]. Abdelbary and Fahmy also observed a downward trend in particle diameter with increased surfactant concentration [27]. This phenomenon can be explained by surfactant‐induced reduction of interfacial tension between lipid and aqueous phases, facilitating formation of smaller emulsion droplets and preventing particle aggregation through steric stabilization [28, 29]. Low surfactant concentrations fail to fully coat lipid droplets, promoting aggregation and increased particle size [30]. Similar size reductions with increased surfactant have been reported by Sahu et al. [31], Ahmad et al. [32], and Dang et al. [25]. Kamel et al. further confirmed that increasing poloxamer surfactant concentration reduces SLN size [33].

Our results revealed a complex relationship between GMS/lecithin lipid ratio and particle size: Increasing the ratio from 0.25 to 1.0 decreased size, while further increase from 1.0 to 4.0 caused size growth. This aligns with findings by Afra et al. [30], Shah et al. [34], and Sahu et al. [31], who associated increased lipid content with larger particle size, likely due to elevated suspension viscosity reducing shear efficiency and relatively lower surfactant coverage [35]. Conversely, Demirbilek et al. reported decreased particle size with increasing lipid ratio [36]. Rosita et al. and Banala et al. similarly found increased particle size with higher glyceryl monostearate content [22, 37].

PdI, indicating particle size distribution uniformity, is another critical optimization parameter; low PdI values denote monodisperse populations [32, 33, 38]. We observed that increasing the GMS/lecithin ratio from 0.25 to 3.0 decreased PdI, consistent with Afra et al. [30]. However, a further increase in the lipid ratio caused PdI to rise, possibly due to increased viscosity promoting particle coalescence and aggregation [34]. Banala et al. reported similar PdI increases with elevated lipid content [37].

Surfactant ratio also affects PdI, with our results showing a decline in PdI with increasing Tween 80 concentration. This agrees with Darabi et al. [39], Kaur et al. [40], and Surve et al. [41], who demonstrated decreased PdI with higher surfactant content, attributed to reduced interfacial tension and improved homogeneity. Contrastingly, some studies reported increased PdI at higher surfactant levels due to the formation of unevenly sized nanoparticles [32].

Zeta potential is a key indicator of nanoparticle suspension stability [42]. Our SLNs exhibited a zeta potential of approximately −22.6 ± 3.3 mV, reflecting substantial stability through electrostatic repulsion, preventing aggregation during storage [30].

Freeze drying (lyophilization) was employed to enhance nanoparticle stability. Postlyophilization, zeta potential increased without significant size changes, indicating preservation of nanoparticle structure and prevention of Ostwald ripening and hydrolytic degradation [43, 44].

The release profile of bee pollen flavonoids from SLNs exhibited an initial slow release over the first 2 h without burst release, followed by a continuous and sustained release up to 48 h. This prolonged release is likely due to the encapsulation of flavonoids within the lipid core and their lipophilic nature, resulting in gradual diffusion, consistent with previous reports on SLNs [45, 46].

Many studies have demonstrated the protective effects of bee pollen against hepatotoxicity in animal models [9, 47–52]. However, no prior research has investigated bee pollen′s effects on Pb(CH_3_COO)_2_‐induced liver damage. To our knowledge, this study is the first to demonstrate that both bee pollen and bee pollen ethanolic extract–loaded SLNs exert therapeutic effects on Pb(CH_3_COO)_2_‐induced hepatic injury. Previous research consistently indicates that Pb(CH_3_COO)_2_ induces liver damage primarily via oxidative stress mechanisms [53–59].

The viability of HepG2 cells exposed to Pb(CH_3_COO)_2_ significantly decreased in a dose‐ and time‐dependent manner. The IC_50_ values of Pb(CH_3_COO)_2_ were 20.44 and 10.41 *μ*g/mL at 24 and 48 h, respectively. These findings align with Guermazi et al., who reported a similar dose‐dependent cytotoxicity of Pb(CH_3_COO)_2_ in HepG2 cells [60]. In contrast, exposure to bee pollen extract and bee pollen ethanolic extract–loaded SLNs showed no significant cytotoxicity up to 400 *μ*g/mL at both incubation times. This safety profile is consistent with Doktorovova et al., who noted that SLNs formulated from nontoxic materials typically exhibit negligible cytotoxicity at concentrations below 1000 *μ*g/mL [61]. Nanoparticles with neutral or negative surface charge and smaller sizes generally display lower toxicity and better biocompatibility [62, 63].

Treatment with bee pollen extract before Pb exposure significantly enhanced HepG2 cell viability, protecting against Pb‐induced cytotoxicity. Notably, bee pollen SLNs demonstrated superior cytoprotective effects compared to free bee pollen at all tested concentrations and time points, underscoring the enhanced bioactivity afforded by nanoparticle delivery. These findings highlight the antioxidant and radical‐scavenging properties of bee pollen constituents, as similarly observed by Tsai et al. with caffeic acid pretreatment [64].

Pb exposure not only directly generates ROS but also promotes lipid peroxidation, damaging cell membranes and inducing injury [5, 17, 53, 57, 65–70]. Elevated serum levels of liver enzymes such as AST, ALT, ALP, and LDH are established biomarkers of hepatocellular damage and bile duct obstruction [2, 54, 57, 65]. Consistent with previous studies [1, 4, 66, 71, 72], Pb(CH_3_COO)_2_ exposure significantly increased these enzyme activities, confirming hepatotoxicity. Treatment with bee pollen and bee pollen SLNs markedly normalized these enzyme levels, with SLNs showing superior efficacy.

Flavonoids, abundant in bee pollen, exert antioxidant effects via radical scavenging and metal chelation [73–75]. Correspondingly, Pb exposure significantly increased BLLs, which were effectively reduced by bee pollen, particularly at higher doses of bee pollen SLNs, suggesting enhanced chelating and detoxifying properties of the nanoparticle formulation [12, 18, 48].

Oxidative stress plays a key role in hepatic injury, characterized by increased MDA and NO levels and depletion of antioxidant defenses [76, 77]. Pb(CH_3_COO)_2_ significantly elevated hepatic MDA and NO, indicating lipid peroxidation and reactive nitrogen species generation. Bee pollen and SLNs treatments reduced these markers, with SLNs showing greater protective effects, corroborating Huang et al.′s findings [9].

Antioxidant enzymes—CAT, GPx, and SOD—constitute the primary defense against oxidative damage [5, 9, 70]. Pb exposure significantly suppressed their activities, while bee pollen and SLNs supplementation restored them dose‐dependently, with SLNs again providing superior benefit. These results align with previous reports by Eraslan et al. [48], Abdel Moneim et al. [1], Mohamed et al. [49], and Yıldız et al. [52].

Total thiol content and reduced GSH levels, critical for redox homeostasis, were also decreased by Pb and significantly restored by bee pollen and SLNs, supporting the antioxidant role of bee pollen flavonoids [78–82]. TAC was diminished following Pb intoxication and improved after treatment, particularly with SLNs, confirming the overall enhancement of antioxidant defense [83].

Pb(CH_3_COO)_2_ administration impaired hepatic protein synthesis, reflected by decreased total tissue protein, which was ameliorated by bee pollen and SLNs, consistent with earlier studies [48, 52, 57, 84–86].

Histopathological analysis showed normal liver architecture in controls, while Pb(CH_3_COO)_2_ induced inflammation, congestion, hemorrhage, fibrosis, necrosis, and pyknotic nuclei. Bee pollen coadministration partially ameliorated these changes, whereas bee pollen SLNs substantially reduced all histopathological damages, confirming the enhanced protective effect of nanoparticle delivery [5, 9, 48, 49, 71, 87].

## 5. Conclusion

This study successfully optimized SLNs containing bee pollen by systematically evaluating key formulation factors influencing particle size, PdI, and zeta potential. The optimized SLNs demonstrated favorable physicochemical properties, including small size, uniform size distribution, high EE%, and sustained flavonoid release.

In vivo results confirmed that both bee pollen extract and bee pollen SLNs exert significant hepatoprotective effects against Pb(CH_3_COO)_2_‐induced liver injury, with SLNs exhibiting superior efficacy. Cytotoxicity assays revealed that bee pollen, bee pollen SLNs, and blank SLNs were nontoxic to HepG2 cells, while bee pollen formulations protected cells from Pb‐induced cytotoxicity.

Collectively, these findings suggest that bee pollen ethanolic extract–loaded SLNs represent a promising and effective approach for the prevention and treatment of Pb‐induced hepatotoxicity, offering enhanced bioavailability and antioxidant activity compared to free bee pollen.

## Conflicts of Interest

The authors declare no conflicts of interest.

## Author Contributions

Khashayar Sanemar, Reza Mahjub, and Fatemeh Nouri: data gathering, original draft preparation, and histological analysis. Mojdeh Mohammadi: supervision, data analyses, and reviewing and editing.

## Funding

This research was carried out using financial facilities provided by the Deputy of Research and Technology, Hamadan University of Medical Sciences, Hamadan, Iran, under Grant No. 140110138631.

## Data Availability

The datasets generated and analyzed during the current study are available from the corresponding author upon reasonable request.
